# Neoadjuvant chemoradiotherapy in patients with unresectable locally advanced sigmoid colon cancer: clinical feasibility and outcome

**DOI:** 10.1186/s13014-021-01823-4

**Published:** 2021-05-24

**Authors:** Shao-Qing Niu, Rong-Zhen Li, Yan Yuan, Wei-Hao Xie, Qiao-Xuan Wang, Hui Chang, Zhen-Hai Lu, Pei-Rong Ding, Li-Ren Li, Xiao-Jun Wu, Zhi-Fan Zeng, Wei-Wei Xiao, Yuan-Hong Gao

**Affiliations:** 1 State Key Laboratory of Oncology in South China, Collaborative Innovation Center for Cancer Medicine, Guangzhou, People’s Republic of China; 2 Department of Radiation Oncology, Sun Yat-sen University Cancer Center, 651 Dongfeng Rd East, Guangzhou, 510060 People’s Republic of China; 3 Department of Radiation Oncology, The First Affiliated Hospital of Sun Yat-sen University, Guangzhou, Guangdong People’s Republic of China; 4 Department of Colorectal Surgery, Sun Yat-sen University Cancer Center, Guangzhou, People’s Republic of China

**Keywords:** Sigmoid colon cancer, Neoadjuvant chemoradiotherapy, Down staging, Pathological complete response, Organ preservation

## Abstract

**Background:**

Patients with locally advanced sigmoid colon cancer (LASCC) have limited treatment options and a dismal prognosis with poor quality of life. This retrospective study aimed to further evaluate the feasibility and efficacy of neoadjuvant chemoradiotherapy (NACRT) followed by surgery as treatment for select patients with unresectable LASCC.

**Methods:**

We studied patients with unresectable LASCC who received NACRT between November 2010 and April 2019. The NACRT regimen consisted of intensity modulated radiotherapy (IMRT) of 50 Gy to the gross tumor and positive lymphoma node and 45 Gy to the clinical target volume. Capecitabine‑based chemotherapy was administered every 2 (mFOLFOX6) or 3 weeks (CAPEOX). Surgery was scheduled 6–8 weeks after radiotherapy.

**Results:**

Seventy‑two patients were enrolled in this study. Patients had a regular follow-up (median, 41.1 months; range, 8.3–116.5 months). Seventy‑one patients completed NACRT, and sixty-five completed surgery. Resection with microscopically negative margins (R0 resection) was achieved in 64 patients (88.9%). Pathologic complete response was observed in 15 patients (23.1%), and multivisceral resection was necessary in 38 patients (58.3%). The cumulative probability of 3-year overall survival (OS) and progression-free survival (PFS) were 75.8 and 70.7%, respectively.

**Conclusions:**

For patients with unresectable LASCC, neoadjuvant chemoradiotherapy is feasible, surgery can be performed safely and may result in increased survival and organ preservation rates.

## Background

Colorectal cancer is the third most common malignancy and the third leading cause of cancer-related death worldwide [1]. Approximately 15% of patients present with locally advanced tumor (T4 stage), and if the tumor directly invades other organs or structures, multivisceral resection (MVR) is required [2]. Despite application of multiple treatment strategies, patients with locally advanced colon carcinoma (LACC) still have poor prognoses [3–7].

Neoadjuvant chemoradiotherapy (NACRT) has been established as standard therapy for local advanced rectal cancer (LARC) [8] and may reduce local recurrence, but this has not been elucidated in colon cancer. Results of existing series studies of colon cancer show that neo-chemoradiotherapy can be beneficial for selected unresectable LACCs [9–14]. Our previous study[10] also proved that preoperative chemoradiotherapy and surgery can be performed safely and may result in an increased survival rate in patients with locally advanced sigmoid colon cancer (LASCC). In this study, we expand the sample size and prolong the follow-up time, and we described the treatment results of the adoption of NACRT for unresectable LASCC patients.

## Methods and materials

### Patient selection

Patients with pathologically diagnosed and unresectable LASCC in our hospital between November 2010 and April 2019 were enrolled. This was an observational study approved by our institutional medical ethics committee (B2020-174-01).

Patients with LASCC (defined as the primary tumor having an inferior margin > 15 cm from the anal verge) were selected to undergo NACRT on a case-by-case basis through multidisciplinary team consultation. This study had the following inclusion criteria: (1) curative resection was impossible due to preoperative imaging examinations showing that the tumor extensively involved adjacent organs/structures or involved multiple lymph node metastases, making radical resection difficult to achieve (47 patients); (2) curative resection was deemed impossible after exploratory laparotomy (25 patients). Patients with the following criteria were excluded: (1) patients with uncontrolled medical conditions (e.g., hypertension, diabetes, heart failure, or psychiatric disease); (2) prior history of other malignancies. Before treatment, written informed consent was obtained from all patients.

### Treatment procedure details

Radiotherapy (RT) was delivered using IMRT with 6 MV photon beams, and all plans were calculated using the Eclipse or Monaco system. Median radiation doses were 50 Gy (range: 45.0–54.0 Gy) for gross tumor volume (GTV) and positive lymph node, and 45 Gy (46.0–56.0 Gy) for clinical target volume (CTV) with conventional segmentation. GTV was defined as the macroscopic tumor and involved regional lymph nodes shown on imaging studies and physical examination before treatment. CTV as defined as GTV with a cranio-caudal margin of 2–3 cm, sigmoid mesocolon and lymphatic drainage regions. If adjacent structures were involved, a further 1.5 cm isotropic margin into the involved structures and the ischiorectal fossa was included to account for microscopic disease and possible implantation metastases to the pelvic floor.

The neoadjuvant chemotherapy regimen included the CAPEOX regimen (Oxaliplatin 130 mg/m^2^ IV over 2 h, day1; Capecitabine 1000 mg/m^2^ twice daily PO for 14 days, repeat every 3 weeks) for the majority of patients who received a first diagnosis (89.1%), and the mFOLFOX6 regimen (Oxaliplatin 85 mg/m^2^ IV, day1; Leucovorin 400 mg/m^2^ IV, day 1; 5-Fu 400 mg/m^2^ IV bolus on day 1, then 2400 mg/m^2^ over 46–48 h IV continuous infusion; repeat every 2 weeks) which was administered to patients who were unable to receive oral medications because they presented with symptoms of an intestinal obstruction. Elderly (> 70 years) patients were treated with capecitabine monotherapy. The median cycle of neoadjuvant chemotherapy is 3 (range: 1–5 cycles). Sixty-four patients received postoperative adjuvant chemotherapy and the median cycle is 4 (range 1–9 cycles). The adjuvant chemotherapy regimen was determined according to the efficacy of previous chemotherapy, the performance status (PS) score and medical complications. If the efficacy of the previous chemotherapy regimen was partial response (PR) or stable disease (SD), the previous regimen would be continued. If the efficacy was local tumor progression without distant metastasis (DM), an oxaliplatin regimen or irinotecan-based regimen was used alternately. If the patient’s previous chemotherapy intensity was strong and PS score was 2, then the patient would receive capecitabine alone. The postoperative adjuvant chemotherapy consisted of a capecitabine-based regimen, including CapeOX and mFOLFOX6.

Surgery was scheduled 6–8 weeks after RT. All imaging and blood tests were repeated before surgery. When tumor infiltration or adhesion to adjacent organs was detected intraoperatively, MVR was required.

### Response, toxicity and complications

Acute and late adverse events were graded according to the Common Terminology Criteria for Adverse Events (version 4.03). Surgical complications were assessed according to the Clavien-Dindo classification [15].

### Follow-up

Outpatient follow-up visits were performed every 3 months during the first 2 years after treatment, semiannually in the subsequent 3 years, and then yearly thereafter. Patients were followed up by outpatient interview or telephone until death or through May 31, 2020.

### Statistical analysis

Statistical analysis was performed using Statistical Product and Service Solutions software for Windows (SPSS Inc., Version 25, Chicago, IL). Kaplan–Meier curves were used to calculate overall survival (OS), progression-free survival (PFS), and local control (LC). PFS was defined as freedom of local, regional and distant failure from the date of diagnosis with biopsy confirmation to date of first documented relapse. Patients who were alive at last follow-up and progress free were censored. Primary end points were OS and PFS. Secondary end points included tumor response grade (TRG) and the rate of R0.

## Results

### Characteristics and compliance

Between November 2010 and April 2019, a total of 72 patients were enrolled in this study. The mean age was 56 (range: 29–79) years old, and 75% (n = 54) were males. According to the 8th edition of the Union for American Joint Cancer Committee (AJCC) TNM staging system, 9 patients were diagnosed with T3 stage, 5 with T4a and 58 with T4b disease. Three quarters of pathologic diagnoses were moderately differentiated adenocarcinoma. The most commonly involved organs were bladder (62.5%), abdominal/pelvic wall (25.0%), small intestine (12.5%) and ureter (11.1%). Among the 45 patients with bladder involvement, six were proved by cystoscopy and the rest by CT and/or MRI and irritation signs of the bladder (e.g. hematuria, urgent urination, frequent micturition, odynuria). Clinical and treatment characteristics of study patients are presented in Table 1.

**Table 1:** Baseline clinicopathologic characteristics of the 72 patients with unresectable local advance sigmoid colon cancer (LASCC)

Characteristic	No. (%)	
*Age*		
≤ 65	58 (80.6)	
> 65	14 (19.4)	
*Gender*		
Male	54 (75.0)	
Female	18 (25.0)	
*cT stage*		
T3	9 (12.5)	
T4a	5 (6.9)	
T4b	58 (80.6)	
*cN stage*		
N0	1 (1.4)	
N1	25 (34.7)	
N2	46 (63.9)	
*Clinical stage*		
IIc	1 (1.4)	
IIIb	15 (20.8)	
IIIc	55 (76.4)	
IV	1 (1.4)	
*Tumor differentiation*		
High	15 (20.8)	
Moderate	54 (75.0)	
Low	3 (4.2)	
*Involved organ*		
Bladder	45 (62.5)	
Ureter	8 (11.1)	
Abdominal/Pelvic wall	18 (25.0)	
Small intestine	9 (12.5)	
*CEA*		
≤ 5 ng/ml	30 (41.7)	
> 5 ng/ml	35 (48.6)	
Unknown	7 (9.7)	
*Bladder fistula/perforation*		
Yes	14 (19.4)	
No	58 (80.6)	
*Intestinal obstruction*		
Yes	13 (18.1)	
No	59 (81.9)	
*Family history*		
Yes	12 (16.7)	
No	60 (83.3)	
*MMR*		
dMMR	6 (8.3)	
pMMR	40 (55.6)	
Unknown	26 (36.1)	
*KPS*		
≥ 90	58 (80.6)	
< 90	14 (19.4)	

KPS, Karnofsky Performance Status; BMI, Body Mass Index; cT stage, clinical T stage; cN stage, clinical N stage; MMR, mismatch repair phenotype

### Short-term clinical efficacy

**Fig. 1:**
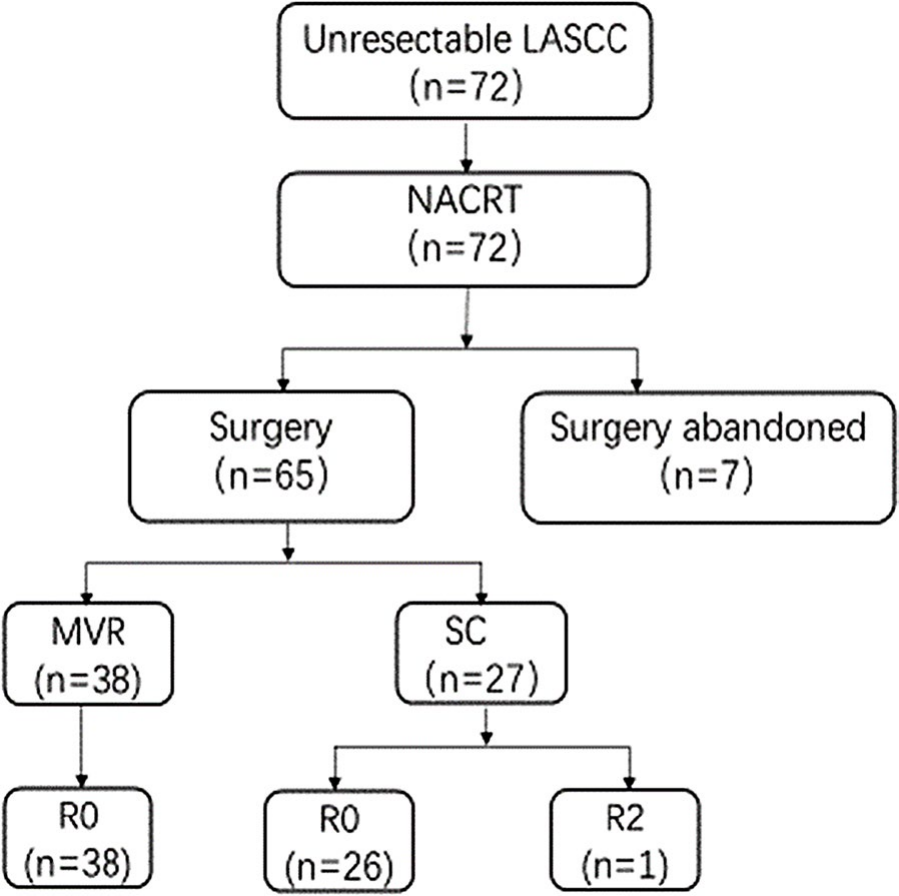
Treatment flow chart. MVR: multivisceral resection; SC: simple colectomy

After NACRT, 65 patients with initially unresectable tumors were successfully transformed to operable, while tumors in 7 patients failed to reach this criterion. Among the 65 patients undergoing surgery, 64 (64/72, 88.89%) patients who received radical surgical resection with negative margins (R0), and one exhibited tumor residue (R2) (Fig. 1). Fifteen patients (23.1%) experienced pathological complete remission (pCR) after NACRT. As the TRG assessment after NACRT, 11 patients (25.6%) showed TRG1, 12 patients (27.9%) showed TRG2, 13 patients (30.2%) showed TRG3 and 7 patients (16.3%) showed TRG4.

Among the 45 patients with bladder invasion before treatment, only two received complete bladder excision, 21 received partial bladder excision, and 15 patients’ bladders were completely preserved (the remains 7 patients abandoned surgery). Surgical results and pathological findings are detailed in Table 2.

**Table 2:** Treatment outcomes of surgery and pathological findings in 72 patients with LASCC

Outcomes	No. (%)
*Surgery situation*^$^	
R0	64 (88.9)
R2	1 (1.4)
Abandoned	7 (9.7)
*pT stage*	
T0	10 (13.9)
T1	2 (2.8)
T2	7 (9.7)
T3	26 (36.1)
T4a	6 (8.3)
T4b	14 (19.4)
*pN stage*	
N0	62 (86.1)
N1	3 (4.2)
N2	0
*Downstage T*	
Yes	54 (75.0)
No	11 (15.3)
*Downstage N*	
Yes	63 (87.5)
No	2 (2.8)
*Downstage*	
Yes	62 (95.4)
No	3 (4.6)
*MVR*	
Yes	38 (58.5)
No	27 (41.5)
*pCR*	
Yes	15 (23.1)
No	50 (76.9)
*TRG*^#^	
1	11 (25.6)
2	12 (27.9)
3	13 (30.2)
4	7 (16.3)

pT stage, postoperative pathology T stage; pN stage, postoperative pathology N stage; MVR, multivisceral resection; pCR, Pathologic complete remission; NA: not available; TRG: tumor regression grade

^$^Seven patients abandoned surgery

^#^Twenty-nine patients was unavailable

### Long-term survival

**Fig. 2:**
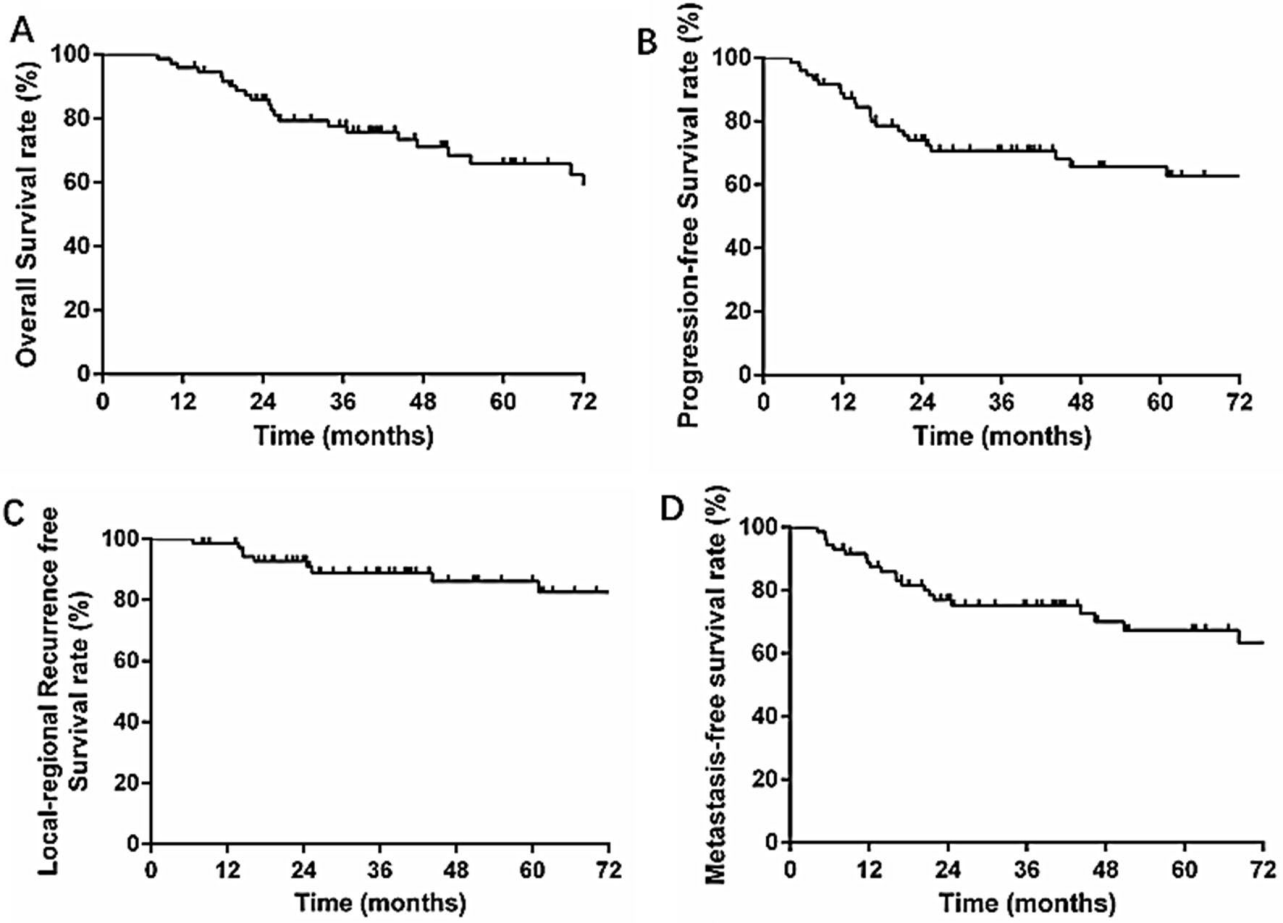
Survival curves of the 72 patients with unresectable LASCC. **A** Overall survival (OS), **B** progression-free survival (PFS), **C** locoregional recurrence‑free survival (RFS), **D** metastasis‑free survival(MFS)survival curves of the 72 patients with unresectable LASCC. The estimated 3-year OS, PFS, RFS and MFS were 75.8%, 70.7%, 89.0%, and 75.2%, respectively

Median follow-up of surviving patients was 41.1 months (range, 8.3–116.5 months) in the entire group. The estimated 3-year OS, PFS, recurrence-free survival (RFS) and metastasis-free survival (MFS) were 75.8, 70.7%, 89.0%, and 75.2%, respectively (Fig. 2). During the follow-up period, local–regional recurrence was observed in 9 patients, and the LC rate was 87.5% (63/72).

**Fig. 3:**
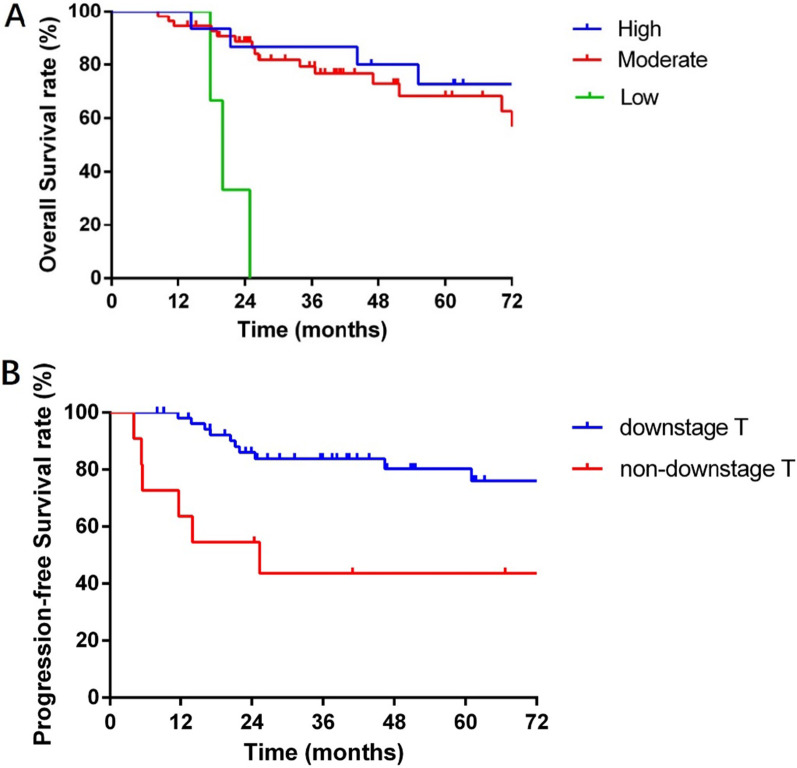
Subgroup analysis of survival. Overall survival by tumor differentiation (**A**) (*p* = 0.003) and progression-free survival by downstaging T (**B**) (*p* = 0.027) in patients with unresectable LASCC with neoadjuvant chemoradiotherapy and surgery

In univariate analysis, non-R0 resection, non-downstaging T, postsurgical pathology N stage (N1), postsurgical pathology T stage (T4a–T4b), low differentiation and perineurium invasion (PNI) were significantly associated with poorer OS, while non-R0 resection, no pathological complete remission (non-pCR), non-downstaging T, postsurgical pathology T stage (T4a–T4b) and PNI were associated with reduced PFS (*p* < 0.05) (Table 3). In multivariate analysis, differentiation remained an independent prognostic factor for OS (Fig. 3A). Meanwhile, downstaging T was an independent prognostic factor for PFS (Fig. 3B).

**Table 3:** Univariate and multivariable Cox analysis of prognostic factors for overall survival and progression free survival in 72 patients with unresectable sigmoid colon cancer (LASCC) treated with neoadjuvant chemoradiotherapy and surgery

	Univariate analysis		Multivariable analysis	
	*p*	HR (95% CI)	*p*	HR (95% CI)
*Overall survival variable*				
R0 resection (R0 vs. non-R0)	< 0.001	0.292 (0.184–0.465)	0.973	0.937 (0.020–42.855)
pCR (pCR vs. non-pCR)	0.172	2.342 (0.690–7.946)	0.339	0.194 (0.007–5.597)
Down T stage (yes vs. non)	0.044	3.031 (1.033–8.894)	0.426	2.021 (0.358–11.419)
pN Stage group (pN0 vs. pN1)	0.037	5.234 (1.105–24.800)	0.601	2.845 (0.057-142.977)
pT Stage group (pT0-T3 vs. pT4a-4b)	0.023	2.678 (1.144–6.272)	0.634	0.659 (0.118–3.664)
Differentiation	0.001	0.062 (0.012–0.319)	0.003	36.443 (3.500–379.429)
PNI (yes vs. non)	0.011	4.138 (1.377–12.435)	0.875	1.205 (0.119–12.166)
*Progress-free survival variable*				
R0 resection (R0 vs. non-R0)	< 0.001	0.343 (0.219–0.538)	0.232	0.091 (0.002–4.629)
pCR (pCR vs. non-pCR)	0.035	3.697 (0.865–15.806)	0.991	497,046.093 (0.000–infinity)
Down T stage (yes vs. non)	0.006	4.109 (1.490–11.333)	0.027	6.095 (1.228–30.253)
pT Stage group (pT0-T3 vs. pT4a-4b)	0.010	2.988 (1.293–6.907)	0.797	0.818 (0.177–3.786)
Differentiation	0.111	2.292 (0.827–6.356)	0.130	4.246 (0.653–27.599)
PNI (yes vs. non)	0.015	3.883 (1.300-11.597)	0.251	3.108 (0.448–21.581)

Within follow-up period, 9 patients (9/72, 12.5%) experienced recurrence. Frequent recurrence sites were observed in bladder (n = 2), ureter (n = 2), anastomotic stoma (n = 2), peritoneum (n = 2) and iliac lymph nodes (n = 1).

### Treatment-related toxicity

Treatment toxicities were assessed according to CTCAE criteria version 4.03 as shown in Table 4. The most common NACRT-related toxicities were grade 1 to 2 myelosuppression (88.9%), mucositis/dermatitis (97.2%) and gastrointestinal (GI) toxicities (93.1%). Four patients developed intestinal obstruction during NACRT. Only one patient failed to complete the radiation course due to tumor rupture and underwent emergency surgery. Among the 65 patients who underwent surgery, grade 3/4 Clavien-Dindo postsurgical complications were observed in 5 cases (7.7%) and anastomotic leakage in three patients (4.6%).

**Table 4:** NACRT toxicities and surgical complications in the 72 patients with unresectable local advance sigmoid colon cancer (LASCC)

Adverse effects	No. (%)
*Myelosuppression*	
Grade 0–2	64 (88.9)
Grade 3–4	8 (11.1)
*Mucositis/dermatitis*	
Grade 0–2	70 (97.2)
Grade 3–4	2 (2.8)
*GI toxicities*	
Grade 0–2	67 (93.1)
Grade 3–4	5 (6.9)
*Intestinal obstruction*	
Yes	4 (5.6)
No	68 (94.4)
*Anastomotic leakage*^#^	
Yes	3 (4.6)
No	62 (95.4)

GI: gastrointestinal, MVR: multivisceral resection; SC: simple colectomy

^#^Including 7 cases who abandoned surgery

## Discussion

Although NACRT is the standard treatment for LARC, its place in the management of sigmoid colon cancer has yet to be defined. There are several single or very small sample size case reports about utilizing NACRT for sigmoid colon cancer [12, 16–18]. Cukier et al. [18] retrospectively reviewed 33 patients with potentially resectable, non-metastatic primary LACC who received neoadjuvant CRT, and all patients had R0 resection. The rates of pCR and 3-year OS were 3 and 85.9%, respectively. Our previous work [10] also revealed promising clinical outcomes and mild side effects in response to NACRT in LASCC, in which all 21 LASCC patients (100%) with locally unresectable disease attained resectable disease, including 14 patients (66.7%) who received a simple colectomy and 7 patients (22.2%) who were in need of MVR. The rates of pCR and 3-year OS were 38.1 and 95.2%, respectively.

MVR is the recommended surgical treatment for LACC [19]. Mohan et al. [20] found MVR to be associated with the best chance of long term survival when clear margins are achieved, and R0 resection was the strongest factor associated with long-term survival when analyzing 22 studies comprising 1575 patients from 1995 to 2012. Therefore, whether LACC patients could be successfully transformed from unresectable to resectable status is crucial for the goal of cure. NACRT provides patients with unresectable LASCC a choice to improve resectability and survival. Ideal treatment results were also seen in our study in 65 patients (90.3%) who successfully transformed to resectable status. After NACRT, 53.5% patients achieved TRG1 and TRG2, and 64 patients achieved R0 resection (88.9%) in our study. Compared with the result of FOxTROT trial [21], which showed that 31% patients in preoperative chemotherapy group achieved moderate or greater TRG, the relative better TRG in our study may be due to the addition of RT to chemotherapy. RT may contribute to better tumor regression, as observed in patients with LARC. The higher R0 resection rate of LASCC in our study also translated to long-term survival benefit.

In our study, NACRT achieved satisfactory clinical outcomes. Sixty-five patients received surgical resection after NACRT. According to postoperative pathological results, 62 patients experienced down-staging, and the pCR rate was as high as 23.1%. T stage was down-graded in 54 patients (75.0%), and the N stage was down-graded in 63 cases (87.5%). In fact, when followed up for a median period of 41.1 months, the 3-year OS and PFS were 75.8 and 70.7%, respectively, which is comparable to results recorded in the literature [9, 10, 22]. In this study, the 3-year recurrence rate was 12.5%, while the 2-year rate of relapse or persistent disease in neoadjuvant chemotherapy group was 14% in FOxTROT trial. In another study, patients with LACC who received neoadjuvant triplet chemotherapy regimen, the 2-year recurrence rate is 26.1% [23]. The above results showed that NACRT could get better LR compared with neoadjuvant chemotherapy. As to survival, Zhou et al. reported 2-year DFS rate was 73.9% in LACC patients who adpoted neoadjuvant chemotherapy [23], which was comparable with this study with a 3-year PFS of 75.8% after NACRT.

In addition to improving the prognosis, NACRT ameliorates organ preservation during surgery. The bladder and small intestine are the most commonly affected organs in LASCC, which are most commonly removed in MVR. In this study, 45 patients (62.5%) exhibited bladder invasion before treatment. Among the 65 patients receiving surgery, only 21 cases (32.3%) received partial cystectomy, while two received total cystectomy. Owing to NACRT, 36 patients (36/45, 80%) retained bladder function. Therefore, NACRT improved quality of life in these patients by preserving important organs.

Acute toxicities in response to NACRT were mild. Myelosuppression and radiodermatitis/mucositis were the most common adverse events. For myelosuppression, grade 1–2 incidence was 88.9%, and grade 3–4 was 11.1% in this study. Zhou et al. [23] reported the rate of grade 3–4 toxicities was up to 56.5% in patients who received FOLFOXIRI regimen neoadjuvant chemotherapy. The relative mild myelosuppression is associated the two chemotherapeutic agents adopted in majority of patients enrolled in this study. The incidences of grade 1–2 and grade 3–4 mucositis and dermatitis were 97.2 and 2.8%, respectively. The adverse events in our study were similar to previous literature report with preoperative chemoradiotherapy in LASCC [13]. In this study, five patients (6.9%) experienced grade 3–4 gastrointestinal reactions, which was comparable with FOxTROT trial result with 7% patients in preoperative chemotherapy group had grade 3 or worse gastrointestinal toxicity [21]. This result showed that the radiotherapy didn’t increase the gastrointestinal toxicity. Besides, three patients (4.6%) experienced anastomotic leakage, which was comparable with the FOxTROT trial result (5%), which means RT did not increase the incidence of anastomotic leakage.

There are several limitations to this study. First, the sample size was small, and the median follow-up period of 41.1 months was rather short. Second, this study was a retrospective study and we need randomized controlled trial to validate the results of NACRT in patients with LASCC.

## Conclusions

NACRT is feasible in patients with unresectable LASCC, and surgery can be performed safely and may result in increased survival and organ preservation rates.

## Data Availability

The datasets analyzed during the current study available from the corresponding author on reasonable request.

## Abbreviations

LASCC: Locally advanced sigmoid colon cancer
NACRT: Neoadjuvant chemoradiotherapy
MVR: Multivisceral resection
IMRT: Intensity modulated radiotherapy
R0 resection: Microscopically negative margins
LACC: Local advanced colon cancer
LARC: Local advanced rectal cancer
RT: Radiotherapy
GTV: Gross tumor volume
CTV: Clinical target volume
PS: Performance status
PR: Partial response
SD: Stable disease
DM: Distant metastasis
LC: Local control
OS: Overall survival
PFS: Progression-free survival
pCR: Pathological complete remission
TRG: The tumor regression grading
AJCC: American Joint Cancer Committee
RFS: Recurrence-free survival
MFS: Metastasis-free survival
MMR: Mismatch repair phenotype
PNI: Perineurium invasion
